# Joint trajectory, transmission time and power optimization for multi-UAV data collecting system

**DOI:** 10.1016/j.heliyon.2024.e26627

**Published:** 2024-02-23

**Authors:** Qing Cai, Zheng Tang, Chuan Liu

**Affiliations:** a Yunnan Zhongheng Construction Co., Ltd, 15 Building of Jin Shangjun Garden, Panlong District, Kunming, Yunnan, 650224, China; b School of Information Engineering, Wuhan University of Technology, Wuhan, Hubei, 430070, China

**Keywords:** Multiple UAVs, Data collection, Trajectory optimization, Collection scheduling, Transmit power

## Abstract

Unmanned aerial vehicles (UAVs) have been generally applied in the field of communication due to their small size, flexible mobility, and convenient deployment. As a mobile base station, the UAV node can quickly establish a line-of-sight link with the ground node, thereby improving communication performance. In this paper, we study a multi-UAV assisted data collecting system. Specifically, in the case of limited system energy consumption, UAV flight energy consumption and ground node data transmission energy consumption are considered as an general limitation, and considering the channel interference between nodes, a multi-UAV assisted data collection model is studied. An non-convex problem that maximizes the minimum amount of data collected from ground nodes is further formulated. Since the original optimization problem is non-convex that difficult to solve directly, the problem is first decomposed into four sub-problems, and then the solution of each sub-problem is obtained by using successive convex approximation and block coordinate descent method. Finally, based on the solution of the four subproblems, an iterative algorithm for joint optimization of data transmission planning, transmission power, UAV trajectory and mission time is proposed. Simulation experiments show that the proposed algorithm can obtain more transmission data than the baseline algorithms.

## 1 Introduction

Because of the benefits of small size, flexible movement, convenient deployment, unmanned aerial vehicle (UAVs) have been generally applied in many fields, such as data collection [1], crowd detection [2], and emergency rescue [3]. With the help of technologies such as global cellular networks and satellite communications, drones located in any place can be controlled at any time to complete tasks under the condition of communication network coverage.

Compared with the traditional wireless communication network, the UAV communication network has the advantages of fast data transmission speed, high stability and security, and small network delay. The UAV can be linked by the cellular network to achieve beyond-the-horizon control of the UAV. The UAV communication network is controlled by line-of-sight (LoS) links, which can achieve better communication quality. For example, in a natural disaster scenario, where the basic communication facilities are broken, multiple UAVs set up an independent communication system in the air to restore communication in the disaster area [4]. In the face of Internet of Things (IoT) network scenarios where a great amount of ground nodes (GNs) are deployed, UAVs can quickly and efficiently perform data collection tasks [5].

UAV technology has become a hot research topic and has been widely used in many fields. However, UAVs have some limitations and challenges in practical applications. Firstly, since the UAV is far away from ground nodes, it may not be able to collect enough data and meet the communication requirements. Obviously, the flight efficiency and task execution capability of UAVs largely depend on its flight trajectory. Reasonable trajectory planning can not only ensure the safety of the flight process, but also help UAVs save energy. At the same time, trajectory planning is of great value for UAVs to deal with messy obstacles in complex urban environments and other scenarios [6]. Secondly, using the movement of the UAV instead of relay forwarding for communication will inevitably bring about greater data acquisition delay, making the data acquisition cycle longer and reducing the availability of the system [7]. Therefore, it is important to minimize the completion time required for each data collection task. At the same time, in applications such as intelligent transportation and health monitoring, it is necessary to transmit the generated status information to the destination as soon as possible for online data analysis and decision-making. Outdated information can lead to wrong controls and even catastrophe. Completing data collection as quickly as possible also allows more time for data processing and decision-making [8]. Therefore, it is very important to minimize the task completion time in the UAV data collection system. In addition, due to the limitation of actual physical conditions, the onboard energy of UAVs is limited, so that the endurance of UAVs is usually limited. Limited flight time remains one of the bottlenecks for drone communication [9]. It limits the continuous flight and data collection time of the UAV in one mission, so the power limit of the UAV needs to be considered when performing UAV data collection work. Finally, the flight coverage of a single UAV is limited. To enhance the capabilities of UAV when performing tasks, it is important to deploy multiple UAVs in the system, but the introduction of multiple UAVs will bring greater channel interference and reduce the communication quality. Therefore, a reasonable trajectory planning and resource allocation scheme is considered to enhance the competence of data collection. In addition to the above-mentioned problems, it also has limitations such as being susceptible to weather conditions and having limited payload.

Aiming at the limitations and problems of the UAV data collecting system, we constructed a data collection optimization model based on multiple UAVs and multiple ground nodes with limited energy consumption, which includes multi-UAV channel interference, multi-UAV anti-collision trajectory and other sub-models. We optimize the data transmission power to reduce the channel interference problem of multiple UAVs, thereby increasing the data transmission rate. In addition, we use trajectory constraints to construct anti-collision flight trajectories between UAVs, and jointly optimize parameters such as data transmission planning and mission time to solve the problem of maximizing the minimum amount of data collected by multi-UAV systems from ground nodes. By optimizing related variables, the utilization rate of the limited energy of the UAV is improved, so that the UAV can perform and complete more tasks as much as possible under the limited battery energy.

### 1.1 Related work

Using UAVs to complete data collection task has been extensively studied. For example, in [10], the scholars researched the collection problem of a single UAV with multiple GNs under Rician fading channel. Then the authors maximized the minimum communication rate between UAV and GN by optimizing three dimensional (3D) trajectory and GN's communication schedule. Similarly, in [11], the authors studied the issue of data collection for a single UAV under probabilistic line of sight (LoS) channel. Meanwhile, UAVs can also perform data collection tasks in various IoT communication scenarios. For example, in [12], the authors studied the data collection problem of multiple UAVs in an IoT network with wireless power transmission. And the goal of the authors is to maximize the UAV's data collection rate by jointly optimizing UAV's 3D trajectory and communication resources under probabilistic LoS channel. Further, in [13], the authors considered the data collection challenges in an 6G-enabled IoT network. In summary, these studies mostly optimize multiple variables to maximize the UAV's data collection rate, and less consider the data timeliness and energy consumption of UAVs, which are important indicators [14]. That's because, the location distribution of GNs is complex, so it is necessary to consider the timeliness of data generated by GNs. The on-board energy of UAVs is limited, so reasonable energy allocation is required to extend the flight time of UAVs.

Trajectory optimization is an important aspect in the study of UAV data collection. By optimizing the flight path of the UAV, communication delays can be minimized, data transfer rates can be increased, and energy consumption can be reduced. Some studies focus on finding the optimal flight trajectory to meet specific communication requirements by using optimization algorithms, such as genetic algorithm and particle swarm optimization. At present, many scholars have conducted research on the UAV trajectory optimization problem. In [15], the authors studied the UAV trajectory planning problem in the UAV-based environmental monitoring system, and formulated the trajectory planning problem of the unmanned reconnaissance vehicle as an optimization problem for solution. However, it does not consider the channel interference caused by multiple UAVs collecting data. In [16], the authors maximized the average downlink throughput by jointly optimizing UAV trajectory and other constraints, but the study lacked the optimization of task completion time. In [17], the authors studied the method of optimizing the flight trajectory of the UAV by using the tilt steering mechanism at a fixed height. This research mainly considers the trajectory planning in the obstacle environment, and there is a lack of research on the trajectory optimization problem under the constraints of UAV onboard energy and task completion time. In [18], the authors proposed the optimization problem of minimizing the total energy consumption of UAVs by combining area division and UAV trajectory scheduling in order to prolong UAV operation time and related network life. The goal of this study is to minimize energy consumption, but we mainly consider energy consumption as a constraint to maximize the collected data. In [19], the authors proposed a method to jointly optimize 3D UAV trajectories under the constraints of onboard energy, velocity, acceleration, and completion time, so as to maximize the total throughput of users. Although it considers the task completion time and onboard energy, it does not study the task scheduling when multiple UAVs collect data. In summary, the current research on UAV trajectory optimization mainly focuses on limited constraints, including obstacles, energy consumption, etc. Most studies lack the research on the channel interference problem caused by multiple UAVs collecting data. We mainly study the trajectory optimization problem under the joint scheduling of multiple UAVs with various constraints, so as to achieve the maximum amount of data collection.

As for data timeliness, there are two main ways to ensure the data timeliness in UAV data collection problem: Firstly, make the task completion time minimum, so that UAVs collect enough information on GNs as quickly as possible [20], [21], [22], [23], [24], and the other is to consider the age of information (AoI) to ensure the freshness of information [25], [26], [27], [28]. Next, we will first introduce studies related to UAV mission time. In [20], the authors considered a single UAV assisted data collection system in a wireless power transmission scenario. The authors regarded the amount of information that needs to be collected for each GN as a constraint and minimized task completion time by jointly optimizing the UAV's 2D trajectory and GN's communication schedule. In [21], the authors divided the task time minimization: height optimization, 2D trajectory optimization, joint velocity and GN's schedule optimization. In particular, the authors proposed two methods for 2D trajectory optimization: segment-based method and group-based method. While the authors in [22], [23] went a step further, they considered the multiple UAVs data collection problem. Both of them reduced the task completion time for multi-UAVs by jointly optimizing UAV-GN association mechanism and flight path of UAVs. The difference is that in [22], UAVs only seeked the optimal hover points and only collected data at hover points, while [23] further considered the issue of data collection during continuous flight of UAVs. The above research is mostly based on the trajectory discretization method, which discretizes the entire task time into multiple equal time slots. Based on this, [24] studied how to minimize the length of the time slot by jointly optimizing of trajectory, scheduling, and power in the NOMA scenario. Another indicator to measure the data timeliness in UAV data collection problem is AoI, which depicts the elapsed time since the generation of the latest received update. Lower AoI means better freshness of data or less delay of collecting information. In [25], [26], the authors minimized the GN's maximal AoI and average AoI by jointly design GN's association and a single UAV's trajectory, and an algorithm based on dynamic programming and genetic algorithm was designed to find the optimal solution. In [27], the authors considered the problem of minimizing average AoI in the scenario of UAV assisted wireless power transmission. [28] further considered the AoI optimization problem in collaborative data collection problem for multiple UAVs.

At the same time, there are also some studies that consider the issue of energy consumption when in UAV assisted data collection [29], [30], [31], [32], [33], [34]. The energy cost of UAVs mainly contains two types: flight energy consumption and communication energy consumption. In [29], the authors deployed a static UAV network to complete data collection task and minimize transmission power consumption of UAVs. The authors also designed a lightweight algorithm to facilitate online adjustment of UAVs' transmission power. Generally, flight power consumption is greater than communication power consumption, so most studies aim at flight energy consumption. In [30], the authors declared a new 3D fixed wing UAV flight energy consumption model and applied it to data collection problem. For rotor UAVs, the 2D energy consumption model in [35] is commonly used, and [36] also proposed a commonly used 3D energy consumption model. Based on this, UAV data collection issues considering energy consumption mainly take the following forms. First, as in [31], the UAV energy consumption is considered as a constraint to maximize the data collection rate. Second, as in [32], the energy efficient data collection problem is modeled as the ratio of data collection rate to energy consumption. Third, as in [33], [34], the authors minimized UAVs' energy consumption while ensuring the amount of information collected by UAVs. In terms of power optimization, the researchers are working on extending the UAV's flight time by reducing its energy consumption. They explored various strategies, including flight speed control, trajectory optimization and energy recovery. The goal of power optimization is to make the UAV meet the communication needs while minimizing energy consumption. Researchers have conducted many studies on this goal. In [37], the authors considered the change of propulsion power consumption of UAVs at different speeds, and jointly optimized the user scheduling, transmission power and motion parameters of UAVs to maximize the energy efficiency of mobile UAVs. In [38], the authors designed UAV trajectory optimization and transmission power control strategies to maximize its end-to-end throughput. In [39], the authors comprehensively considered the hovering point, power distribution and energy consumption of UAV to improve the energy utilization efficiency in the obstacle environment. In summary, most of the researches combine the trajectory optimization problem and the power optimization problem to improve the flight efficiency and prolong the battery life of UAVs. However, most current research on power optimization considers limited constraints, and there is a lack of research on power optimization under constraints such as task completion time and task scheduling of multiple UAVs. Aiming at these problems, we implement a multi-UAV auxiliary data acquisition system by taking the energy consumption of ground nodes and the flight power consumption of multiple UAVs as overall constraints.

Currently, there is more research focused on single UAV communication and data collection, there are relatively few studies on multi-UAV systems. Compared with single UAV data collection, multi-UAV data collection system has the characteristics of high efficiency and wide range. It makes new optimization metrics need to be introduced into the communication planning and design of UAVs, so the difficulty of solving the optimization problem also increases accordingly. Furthermore, there is no special constraint on the trajectory of a single UAV, while the flight trajectory planning of multiple UAVs needs to meet the anti-collision requirements. The distance between each UAV must always meet its minimum safe distance. It adds complexity to the trajectory planning of multi-UAV systems, resulting in increased computational tasks and algorithm complexity. Another limitation for UAV data collection is the finite capacity of the onboard batteries. It restricts the endurance of UAVs, preventing them from executing data collection tasks and other missions for extended periods. Therefore, under the constraints of limited energy resources, it is very important to plan the trajectory of UAVs reasonably to obtain better mission performance by optimizing relevant variables.

In view of the problems existing in the current research, we studied the construction of a data collection system for multiple UAVs to collect ground node data under the condition of limited energy consumption of the system. We achieve the goal of maximizing the minimum amount of data collected by UAVs from ground nodes by jointly optimizing data transmission planning, UAV trajectory, mission time and other related parameter variables. By collecting as much data as possible from each ground node and reducing the time to complete data collection tasks, the utilization of limited energy is improved.

### 1.2 Motivations and contributions

Inspired by the related work mentioned above, this paper investigates a multi-UAV assisted data acquisition system. Specifically, we deploy multiple UAVs over a relatively large area to get data from multiple ground nodes one by one. The energy consumption of ground nodes and the flight power consumption of multiple UAVs are considered as the overall constraints. The locations of ground nodes are randomly deployed in this area. Our goal is to maximize the data collected from each ground node by jointly optimizing mission time and task scheduling, multiple UAVs trajectories and all nodes transmission power. The contributions of the work are as follows.

• Aiming at the lack of research on multiple UAVs and the limited constraints considered by most of the current research. We study the construction of a multi-UAV assisted data acquisition system model under the condition of constrained system energy consumption. We fully consider constraints such as trajectory optimization, UAV energy consumption, mission time, and communication interference. Then we construct an optimization problem in which multiple UAVs collect data from multiple ground nodes on this basis.
• Aiming at the problem that there will be more mutual interference in the channel between the ground node and the UAV when multiple UAVs perform tasks. We optimize the data transmission power to reduce channel interference and increase the data transmission rate. We further improve the performance of data collection by introducing trajectory constraints to construct collision-proof flight trajectories between UAVs.
• Aiming at the problem where the original optimization problem is non-convex. We decompose it into four sub-problems. Based on the successive convex approximation (SCA) method and block coordinate descent (BCD) technology, we propose a multi-iterative joint optimization algorithm for UAV trajectory, data transmission planning, task time and task scheduling to solve the original optimization problem. The experimental results show that the proposed algorithm has higher data collection efficiency than the three benchmark algorithms and the single UAV case.

The rest information of the paper is structured as follows. Section 2 presents the system model and formulates the problem. In Section 3, we advance an optimization scheme to deal with the original problem. Section 4 verifies the efficiency of the algorithm through numerical simulation. Finally, the paper is concluded in Section 5.

## 2 System model

**Figure 1:**
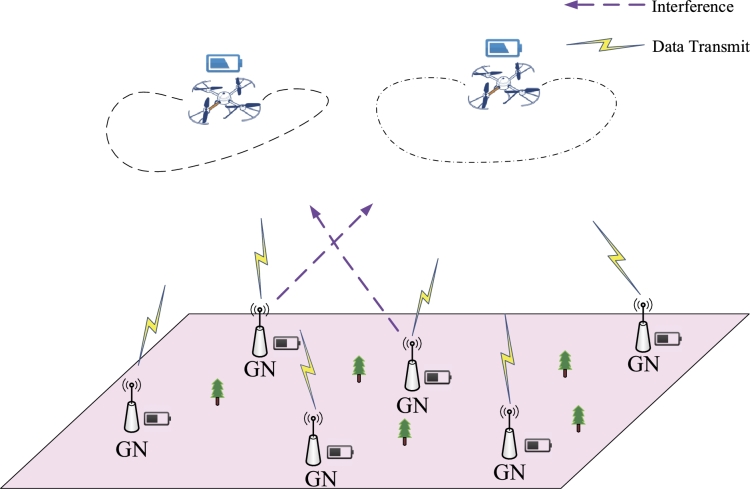
Multi-UAV Data Collection System.

Considering a multiple UAVs data acquisition system as shown in Fig. 1. The model deploys *U* UAVs to acquire information from *K* ground nodes. Introducing a Cartesian 3D coordinate system and ignoring the height, the horizontal coordinate of ground node *k* could be represented by $g_{k}$ = $(x_{k},y_{k})\in R^{2},k=1,\cdots ,K$.

In this model, it is assumed that the UAVs start from the starting point and collect data from all ground nodes along the planned trajectory. Therefore, the total time of the task is expressed as $T_{f}$.

Now we define $V_{max}$ as the maximum speed of UAV. The UAV is flying at a height *H* when collecting data. The trajectory of UAV *u* related to time *t* is represented by $q_{u}[t]=(x_{u}[t],y_{u}[t])\in R^{2},u=1,...,U$. The constraints related to the trajectory of UAVs are now available as follows.

(1) $$q_{u}(0)=q_{u}(T_{f}),\forall u$$

(2) $$\|{\overset{˙}{q}}_{u}[t]\|\leq V_{\max},0\leq t\leq T_{f},\forall u$$

(3) $${\left\|q_{u}[t]-q_{j}[t]\right\|}^{2}\geq d_{s}^{2},0\leq t\leq T_{f},\forall j\neq u$$

where Equation (1) means All UAVs return to starting positions at the end of data collection task. Equation (2) represents the speed constraint of UAVs. Equation (3) means, between any two UAVs, the minimum length is not less than $d_{s}$, so as to ensure flight safety.

Further, we equally divide the total task time $T_{f}$ into (N−1) discrete small slots, i.e., $T_{f}=(N-1)\delta$. *δ* is the distance of time slot. Therefore, the trajectories $q_{u}[t]$ of the UAVs in the horizontal space can be declared discretely as $q_{u}[n]=(x_{u}[n],y_{u}[n])\in R^{2},n=1,\cdots ,N$. It is important to point out that the trajectories of the UAVs need to meet these constraints. Equation (4), Equation (5) and Equation (6) are the discrete forms of Equation (1), Equation (2) and Equation (3) respectively.

(4) $$q_{u}[1]=q_{u}[N],\forall u$$

(5) $${\left\|q_{u}[n+1]-q_{u}[n]\right\|}_{2}\leq D_{\max},n=1,\cdots ,N-1$$

(6) $${\left\|q_{u}[n]-q_{j}[n]\right\|}^{2}\geq d_{s}^{2},\forall n,u,j\neq u$$

where $D_{max}≜\delta V_{max}$. $V_{max}$ is the maximum flight speed of the UAV.

### 2.1 Data collection and interference model

In this system, we use $d_{u,k}[n]$ to represent the distance from GN *k* to the UAV *u* in *n*-th time slot. $d_{u,k}[n]$ can be calculated by Equation (7).

(7) $$d_{u,k}[n]=\sqrt{H^{2}+{\left\|q_{u}[n]-g_{k}\right\|}^{2}},\forall n$$

It is assumed that the channel links between the nodes of the data acquisition system are mainly based on LoS transmission. Then, in term of the free space loss model, the channel gain between the ground node *k* and the UAV *u* could be denoted as Equation (8).

(8) $$h_{u,k}[n]=\beta_{0}d_{u,k}^{-2}[n]=\frac{\beta_{0}}{H^{2}+{\left\|q_{u}[n]-g_{k}\right\|}^{2}},\forall n$$

Where $\beta_{0}$ is the power gain when the reference distance is equal to 1*m*.

Moreover, at *n*-th time slot data transmit power of GN *k* is represented by $p_{k}[n]$ which satisfy $0\leq p_{k}[n]\leq P_{\max}$. $P_{\max}$ is the maximum data transmit power. Since there are multiple ground nodes transmitting data to multiple UAVs at the same time slot, the signal to interference plus noise ratio (SINR) of GN *k* to UAV *u* at the *n*-th time slot can be denoted as Equation (9).

(9) $$\gamma_{u,k}[n]=\frac{p_{k}[n]h_{u,k}[n]}{\sum_{i=1,i\neq k}^{K}p_{i}[n]h_{u,i}[n]+\sigma^{2}}$$

According to Shannon's theorem, if UAV *u* is collecting data from GN *k* in the *n*-th time slot, the data transmit rate $R_{u,k}[n]$ at this time slot can be expressed as Equation (10).

(10) $$R_{u,k}[n]=B\log_{2}\left(1+\gamma_{u,k}[n]\right)=B\log_{2}\left(1+\frac{p_{k}[n]h_{u,k}[n]}{\sum_{i=1,i\neq k}^{K}p_{i}[n]h_{u,i}[n]+\sigma^{2}}\right)$$

In the UAV-assisted data collection system, we introduce a binary variable $b_{u,k}[n]$ to denote the data transmission task. For example, $b_{u,k}[n]=1$ means to execute the task, and $b_{u,k}[n]=0$ means to suspend the task. When UAVs are collecting data, each UAV can only acquire data from one ground node simultaneous and each ground node can only transmit data to one UAV at the same time. Therefore, Therefore, the constraints shown in Equation (11), Equation (12) and Equation (13) are obtained:

(11) $$b_{u,k}[n]\in \{0,1\},\forall u,k,n$$

(12) $$\sum_{k=1}^{K}b_{u,k}[n]\leq 1,\forall u,n$$

(13) $$\sum_{u=1}^{U}b_{u,k}[n]\leq 1,\forall k,n$$

Thus, the achieved rate $R_{k}[n]$ of UAVs collecting ground nodes can be represented as Equation (14).

(14) $$R_{k}[n]=\sum_{u=1}^{U}b_{u,k}[n]R_{u,k}[n]=\sum_{u=1}^{U}b_{u,k}[n]B\log_{2}\left(1+\gamma_{u,k}[n]\right)$$

Therefore, the total sum of data $J_{k}$ collected by UAVs from the GN *k* in the total time $T_{f}$ can be obtained by Equation (15).

(15) $$J_{k}=\sum_{n=1}^{N-1}\delta R_{k}[n]=\sum_{n=1}^{N-1}\sum_{u=1}^{U}\delta b_{u,k}[n]R_{u,k}[n]$$

### 2.2 UAV energy model

Due to the discretization of time, the total energy $E_{GN}$ consumed by all GNs to transmit data can be obtained by accumulating the transmit power of all GNs within the mission time. It can be expressed as Equation (16).

(16) $$E_{GN}=\sum_{k=1}^{K}\sum_{n=1}^{N-1}\delta b_{u,k}[n]p_{k}[n]$$

Based on the existing research results [35], the flight energy consumption of the UAV in the two-dimensional horizontal plane can be denoted as Equation (17).

(17) $$E_{u}(\{v_{u}[n]\})=\sum_{n=1}^{N-1}P_{i}{\left(\sqrt{1+\frac{{\left\|v_{u}[n]\right\|}^{4}}{4v_{0}^{4}}}-\frac{{\left\|v_{u}[n]\right\|}^{2}}{2v_{0}^{2}}\right)}^{1/2}\;\;+\sum_{n=1}^{N-1}\left(P_{0}+\frac{3P_{0}{\left\|v_{u}[n]\right\|}^{2}}{U_{tip}^{2}}+\frac{1}{2}d_{0}\rho sA{\left\|v_{u}[n]\right\|}^{3}\right)$$

where $v_{u}[n]$ represents the UAV flight speed, i.e., $v_{u}[n]≜{\left\|q_{u}[n+1]-q_{u}[n]\right\|}_{2}/\delta \leq V_{\max}$. $P_{0}$ is the induced power and $P_{i}$ is the blade profile power. $U_{tip}$ is the speed of rotor blade. $v_{0}$ means the induced velocity of rotor. Also, the rest of the parameters are environment-dependent constants.

Define $\Delta q_{u}≜{\left\|q_{u}[n+1]-q_{u}[n]\right\|}_{2}\leq \delta V_{\max},n=1,\ldots ,N-1$. Then, the energy consumption of the UAV could be equivalent to the Equation (18).

(18) $$E_{u}(\Delta q_{u})=\sum_{n=1}^{N-1}P_{0}\left(\delta +\frac{3\Delta q_{u}^{2}}{\delta U_{\mathrm{tip}}^{2}}\right)+\sum_{n=1}^{N-1}\frac{1}{2}d_{0}\rho sA\frac{\Delta q_{u}^{3}}{\delta^{2}}\;\;+\sum_{n=1}^{N-1}P_{i}{\left(\sqrt{\delta^{4}+\frac{\Delta q_{u}^{4}}{4v_{0}^{4}}}-\frac{\Delta q_{u}^{2}}{2v_{0}^{2}}\right)}^{1/2}$$

Therefore, the total energy consumption of all UAVs to complete the data collection task is obtained by $E_{U}$. $E_{U}$ can be expressed as Equation (19).

(19) $$E_{U}=\sum_{u=1}^{U}E_{u}$$

### 2.3 Problem formulation

Define $\mu =\min_{k\in K}J_{k}$ and denote $B=\{b_{u,k}[n],\forall u,k,n\}$, $P=\{p_{k}[n],\forall k,n\}$ and $Q=\{q_{u}[n],\forall u,n\}$. The target is to maximize the total amount of data collected by optimizing the binary variable (i.e., **B**), data transmission power (i.e., **P**), UAVs trajectories (i.e., **Q**) and time slot (i.e., *δ*). The above problem could be formulated as Equation (20).

(20a) $$\left(\mathrm{P1}\right)\;:\max_{\left\{B,Q,P,\delta ,\mu \right\}}\;\mu \;s.t.\;\;E_{U}(\{Q\}\delta )+E_{GN}\leq E_{\varepsilon },$$

(20b) $$\sum_{n=1}^{N-1}\sum_{u=1}^{U}\delta b_{u,k}[n]R_{u,k}(\{Q\}\{P\})\geq \mu ,\forall k$$

(20c) $$b_{u,k}[n]\in \{0,1\},\forall u,k,n$$

(20d) $$\sum_{k=1}^{K}b_{u,k}[n]\leq 1,\forall u,n$$

(20e) $$\sum_{u=1}^{u}b_{u,k}[n]\leq 1,\forall k,n$$

(20f) $${\left\|q_{u}[n+1]-q_{u}[n]\right\|}_{2}\leq D_{\max},n=1,\cdots ,N-1$$

(20g) $${\left\|q_{u}[n]-q_{j}[n]\right\|}^{2}\geq d_{s}^{2},\forall n,u,j\neq u$$

(20h) $$q_{u}[1]=q_{u}[N],\forall u,$$

(20i) $$0\leq p_{k}[n]\leq P_{\max},\forall k,n$$

where $E_{\varepsilon }$ is the total energy budget of the network. The energy budget is as follows: UAVs flight energy and GNs transmission information energy. Note that, generally speaking, the energy consumption of UAV flight is much greater than the energy consumption of information transmission of ground nodes.

It should be noted that due to the existence of binary variables and some constraints are non-convex, it is challenging to obtain a solution of the above problem P1. In the following, we will delve into problem P1 and propose an iterative algorithm to solve it based on the block coordinate descent method.

## 3 Proposed solution

The non-convex constraints in problem P1 make it difficult to solve directly. Therefore, we relax the binary variable $b_{u,k}[n]$ into a continuous variable. Secondly we divide the problem into three parts. Finally, we solve the original problem P1 by dealing with the sub-problems of each part.

Aim at solving the original problem, we first relax the binary variables into continuous variables, which can be rewritten as Equation (21).

(21) $$\left(\mathrm{P2}\right)\;:\max_{\left\{B,Q,P,\delta ,\mu \right\}}\;\mu \;s.t.\;\;(20a),(20b),(20d)∼(20i)0\leq b_{u,k}[n]\leq 1,\forall u,k,n$$

**Figure 2:**
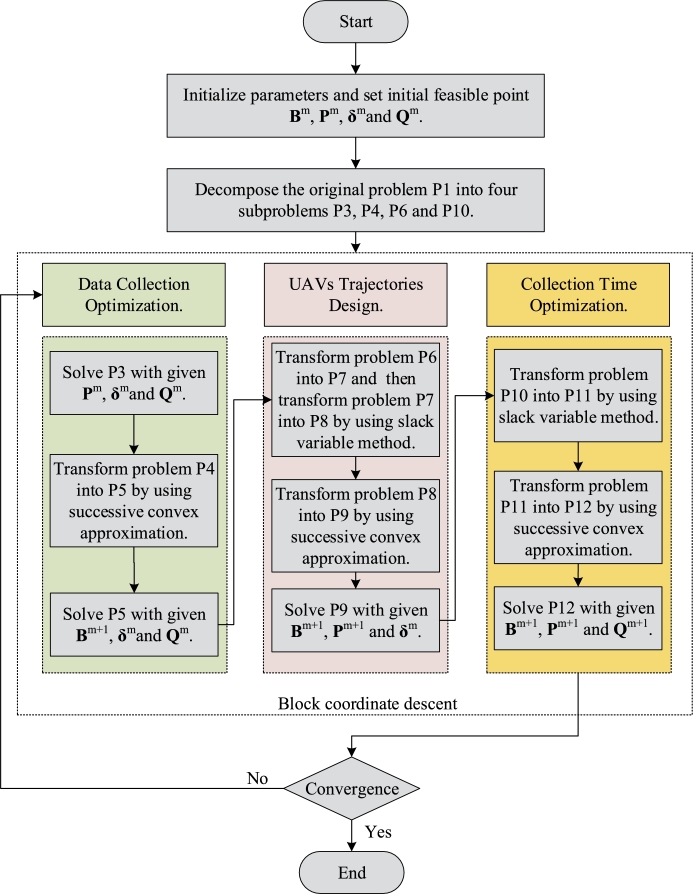
The flow chart of solving problem P2.

In the following research, we will decompose the original problem (P2) into 4 sub-problems and solve them in blocks. The specific flow chart is shown in Fig. 2.

### 3.1 Data collection optimization

This part consists of two aspects: the UAV-GN data acquisition scheduling optimization and the GN's transmission power optimization.

#### 3.1.1 Data collection scheduling

For any given UAVs trajectories $q_{u}[n]$, GN's data transmit power $p_{k}[n]$ and time slot length *δ*, The subproblem of data acquisition scheduling optimization can be written as Equation (22).

(22a) $$\left(\mathrm{P3}\right)\;:\max_{\left\{B,\mu \right\}}\;\mu \;s.t.\;\;E_{U}+\sum_{k=1}^{K}\sum_{n=1}^{N-1}\delta b_{u,k}[n]p_{k}[n]\leq E_{\varepsilon }$$

(22b) $$\sum_{n=1}^{N-1}\sum_{u=1}^{U}\delta b_{u,k}[n]B\log_{2}\left(1+\gamma_{u,k}[n]\right)\geq \mu \forall k$$

(22c) $$0\leq b_{u,k}[n]\leq 1,\forall u,k,n$$

(22d) $$\sum_{k=1}^{K}b_{u,k}[n]\leq 1,\forall u,n$$

(22e) $$\sum_{u=1}^{U}b_{u,k}[n]\leq 1,\forall k,n$$

In problem (P3), we can see that the problem (P3) is a standard convex optimization problem. (P3) which are supposed to be solved through these methods such as CVX.

#### 3.1.2 Transmission power optimization

For any given UAVs trajectories $q_{u}[n]$, data acquisition scheduling $b_{u,k}[n]$ and time slot length *δ*, the GN's transmission power optimization problem can be written as Equation (23).

(23a) $$\left(\mathrm{P4}\right)\;:\max_{\left\{P,\mu \right\}}\;\mu \;s.t.\;\;\sum_{k=1}^{K}\sum_{n=1}^{N-1}\delta b_{u,k}[n]p_{k}[n]+E_{U}\leq E_{\varepsilon }$$

(23b) $$\sum_{n=1}^{N-1}\sum_{u=1}^{U}\delta b_{u,k}[n]BR_{u,k}(\{P\})\geq \mu ,\forall k$$

(23c) $$0\leq p_{k}[n]\leq P_{\max},\forall k,n$$

Problem P4 is a non-convex problem because the constraint (23b) is non-convex. Therefore, we further rewrite $R_{u,k}[n]$ in constraint (23b). According to the logarithmic algorithm, there is the following relationship.

(24) $$R_{u,k}(\{P\})=\log_{2}\left(1+\frac{p_{k}[n]h_{u,k}[n]}{\sum_{i=1,i\neq k}^{K}p_{i}[n]h_{u,i}[n]+\sigma^{2}}\right)=\log_{2}\left(\sum_{i=1}^{K}p_{i}[n]h_{u,i}[n]+\sigma^{2}\right)-{\overset{ˇ}{R}}_{u,k}[n]$$

where

(25) $${\overset{ˇ}{R}}_{u,k}[n]=\log_{2}\left(\sum_{i=1,i\neq k}^{K}p_{i}[n]h_{u,i}[n]+\sigma^{2}\right)$$

It can be found that we rewrite $R_{u,k}[n]$ into the form of the difference between two concave functions (for $p_{k}[n]$).

We can further use the SCA method to handle constraint conditions (23b). Therefore, assume that the given feasible power of GN *k* in the *m*-th iteration is: $P^{m}=\{p_{k}^{m}[n],\forall \;k,n\}$, then, for Equation (25), we have the following inequality relationship at the given feasible Taylor expansion point $p_{i}^{m}[n]$.

(26) $${\overset{ˇ}{R}}_{u,k}[n]=\log_{2}\left(\sum_{i=1,i\neq k}^{K}p_{i}[n]h_{u,i}[n]+\sigma^{2}\right)\leq \sum_{i=1,i\neq k}^{K}D_{u,i}[n]\left(p_{i}[n]-p_{i}^{m}[n]\right)+\log_{2}\left(\sum_{l=1,l\neq k}^{K}p_{l}^{m}[n]h_{u,l}[n]+\sigma^{2}\right)≜{\overset{ˇ}{R}}_{u,k}^{\mathrm{ub}}[n]$$

where $D_{u,i}[n]$ can be calculated by Equation (27).

(27) $$D_{u,i}[n]=\frac{h_{u,i}[n]\log_{2}(e)}{\sum_{j=1,j\neq k}^{K}p_{i}^{m}[n]h_{u,j}[n]+\sigma^{2}},\forall \;u,i,n$$

Finally, after using Equation (26) in Equation (24), problem (P4) can be rewritten as (P5), as shown in Equation (28).

(28a) $$\left(\mathrm{P5}\right)\;:\max_{\left\{P,\mu \right\}}\;\mu \;s.t.\;\;\sum_{k=1}^{K}\sum_{n=1}^{N-1}\delta b_{u,k}[n]p_{k}[n]+E_{U}\leq E_{\varepsilon }$$

(28b) $$\sum_{n=1}^{N-1}\sum_{u=1}^{U}\delta b_{u,k}[n]B(\log_{2}\left(\sum_{i=1}^{K}p_{i}[n]h_{u,i}[n]+\sigma^{2}\right)\;\;\;\;\;-{\overset{ˇ}{R}}_{u,k}^{\mathrm{ub}}[n])\geq \mu ,\forall k$$

(28c) $$0\leq p_{k}[n]\leq P_{\max},\forall k,n$$

We can find that the constraints (28a) and (28c) are linear, and constraint (28b) is convex, which means that P5 is a normal convex optimization problems which is easy to be solved by CVX.

### 3.2 UAVs trajectories design

After processing in the previous subsection, we obtained the sub-optimal data collection scheduling $b_{u,k}[n]$ and GN's transmission power $p_{k}[n]$. Similarly, with any given time slot length *δ*, the subproblem (P6) of Equation (29) can be solved to obtain the optimal all UAVs' trajectories $q_{u}[n]$.

(29a) $$\left(\mathrm{P6}\right)\;:\max_{\left\{Q,\mu \right\}}\;\mu \;s.t.\;\;E_{U}(\{Q\})+E_{GN}\leq E_{\varepsilon }$$

(29b) $$\sum_{n=1}^{N-1}\sum_{u=1}^{U}\delta b_{u,k}[n]BR_{u,k}(\{q_{u}[n]\})\geq \mu ,\forall k$$

(29c) $${\left\|q_{u}[n+1]-q_{u}[n]\right\|}_{2}\leq D_{\max},n=1,\cdots ,N-1$$

(29d) $${\left\|q_{u}[n]-q_{j}[n]\right\|}^{2}\geq d_{s}^{2},\forall n,u,j\neq u$$

(29e) $$q_{u}[1]=q_{u}[N],\forall u$$

In problem (P6), the left part of constraints (29a), (29b), and (29d) are non-convex with respect to $q_{u}[n]$. Therefore problem (P6) requires further processing.

For the convenience of follow-up process, we introduce a slack variable $I=\{I_{u}[n]\geq 0,\forall u\}$ to simplify the third item of $E_{u}(\{Q\})$ in constraint (29a). $I_{u}[n]$ can be expressed as equation (30).

(30) $$I_{u}[n]={\left(\sqrt{\delta^{4}+\frac{\Delta q_{u}^{4}}{4v_{0}^{4}}}-\frac{\Delta q_{u}^{2}}{2v_{0}^{2}}\right)}^{1/2},I_{u}[n]\geq 0\Leftrightarrow \frac{\delta^{4}}{I_{u}{\left[n\right]}^{2}}=I_{u}{\left[n\right]}^{2}+\frac{\Delta q_{u}^{2}}{v_{0}^{2}}$$

Now the flight energy consumption of UAV *u* can be rewritten as Equation (31).

(31) $$E_{u}≜\sum_{n=1}^{N}\left(P_{0}(\delta +\frac{\Delta q_{u}^{2}}{\delta U_{tip}^{2}})+\frac{1}{2}d_{0}\rho sA\frac{\Delta q_{u}^{3}}{\delta^{2}}+P_{i}I_{u}[n]\right)$$

Then problem (P6) can be transformed into problem (P7) of Equation (32) after introducing slack variable $\left\{I_{u}[n]\right\}$.

(32a) $$\left(\mathrm{P7}\right)\;:\max_{\left\{Q,I,\mu \right\}}\;\mu \;s.t.\;\;(29b)∼(29e)\sum_{u=1}^{U}E_{u}(\{q_{u}[n]\},\{I_{u}[n]\})+E_{GN}\leq E_{\varepsilon }$$

(32b) $$I_{u}{\left[n\right]}^{2}+\frac{\Delta q_{u}^{2}}{v_{0}^{2}}\geq \frac{\delta^{4}}{I_{u}{\left[n\right]}^{2}},\forall u,n=1,\ldots ,N-1$$

Constraints (29b), (32a), (29d) and (32b) are still non-convex in problem (P7). Usually, it is not easy to find the optimal solution in polynomial time. Aim to ensure the efficiency of the algorithm, we use methods similar to solving power optimization to solve problem (P7). Then the non-convex part $R_{u,k}[n]$ on the left of constraint (29b) can be transformed into Equation (33).

(33) $$R_{u,k}[n]=\log_{2}\left(1+\frac{\frac{p_{k}[n]\beta_{0}}{H^{2}+||q_{u}[n]-g_{k}|{|}^{2}}}{\sum_{i=1,i\neq k}^{K}\frac{p_{i}[n]\beta_{0}}{H^{2}+||q_{u}[n]-g_{i}|{|}^{2}}+\sigma^{2}}\right)={\overset{ˆ}{R}}_{u,k}[n]-\log_{2}\left(\sum_{i=1,i\neq k}^{K}\frac{p_{i}[n]\beta_{0}}{H^{2}+||q_{u}[n]-g_{i}|{|}^{2}}+\sigma^{2}\right)$$

where

(34) $${\overset{ˆ}{R}}_{u,k}[n]=\log_{2}\left(\sum_{i=1}^{K}\frac{p_{i}[n]\beta_{0}}{H^{2}+||q_{u}[n]-g_{i}|{|}^{2}}+\sigma^{2}\right)$$

Note that Equation (33) is still non-convex with respect to UAVs trajectories $q_{u}[n]$. Then we introduce another slack variables $S=\{S_{u,i}[n]=||q_{u}[n]-g_{i}|{|}^{2},\forall \;i\in K,i\neq k,\forall u,n\}$. Now problem (P7) can be reformulated as problem (P8) as Equation (35), which includes Equation (33), Equation (34) and slack variables $S_{u,i}[n]$.

(35a) $$\left(\mathrm{P8}\right)\;:\max_{\left\{Q,S,I,\mu \right\}}\;\mu \;s.t.\;\;(29c)∼(20e),(32a),(32b)\sum_{n=1}^{N-1}\sum_{u=1}^{U}\delta b_{u,k}[n]B({\overset{ˆ}{R}}_{u,k}[n]-\log_{2}\left(\sum_{i=1,i\neq k}^{K}\frac{p_{i}[n]\beta_{0}}{H^{2}+S_{u,i}[n]}+\sigma^{2}\right))\geq \eta ,\forall k$$

(35b) $$S_{u,i}[n]\leq ||q_{u}[n]-g_{i}|{|}^{2},\;\forall \;u,n,i\neq k$$

(P8) is still non-convex, therefore, to eliminate the non-convexity in constraint (29d), (32b) and (35b), the successive convex optimization technique can be applied. We regard $Q^{m}=\{q_{u}^{m}[n],\forall \;u,n\}$ as the initial UAVs trajectories in the *m*-th iteration which can be selected as Taylor expansion points. In every iteration, the first-order Taylor expansion about ${\overset{ˆ}{R}}_{u,k}[n]$ is applied to obtain a concave lower bound at the given Taylor expansion point, as shown in Equation (36).

(36) $${\overset{ˆ}{R}}_{u,k}[n]=\log_{2}\left(\sum_{i=1}^{K}\frac{p_{i}[n]\beta_{0}}{H^{2}+||q_{u}[n]-g_{i}|{|}^{2}}+\sigma^{2}\right)\geq \sum_{i=1}^{K}-A_{u,i}^{m}[n]\left(||q_{u}[n]-g_{i}|{|}^{2}-||q_{u}^{m}[n]-g_{i}|{|}^{2}\right)\;+B_{u,i}^{m}[n]≜{\overset{ˆ}{R}}_{u,k}^{\mathrm{lb}}[n]$$

where $A_{u,i}^{m}[n]$ and $C_{u,i}^{m}[n]$ are fixed values, and their specific forms are as Equation (37) and Equation (38).

(37) $$A_{u,i}^{m}[n]=\frac{\frac{p_{i}[n]\beta_{0}}{{\left(H^{2}+||q_{u}^{m}[n]-g_{i}|{|}^{2}\right)}^{2}}\log_{2}(e)}{\sum_{l=1}^{K}\frac{p_{l}[n]\beta_{0}}{H^{2}+||q_{u}^{m}[n]-g_{l}|{|}^{2}}+\sigma^{2}},\;\forall \;u,i,n$$

(38) $$C_{u,i}^{m}[n]=\log_{2}\left(\sum_{l=1}^{K}\frac{p_{l}[n]\beta_{0}}{H^{2}+||q_{u}^{m}[n]-g_{l}|{|}^{2}}+\sigma^{2}\right),\forall \;u,i,n$$

Constraints (35b) are non-convex too because $||q_{u}[n]-g_{i}|{|}^{2}$ is convex with respect to $q_{u}[n]$. After using first order Taylor expansion at the feasible expansion point $q_{u}^{m}[n]$, the inequality relationship of Equation (39) can be obtained.

(39) $$||q_{u}[n]-g_{i}|{|}^{2}\geq ||q_{u}^{m}[n]-g_{i}|{|}^{2}+2{\left(q_{u}^{m}[n]-g_{i}\right)}^{T}\times (q_{u}[n]-q_{u}^{m}[n]),\;\forall \;u,n,i\neq k$$

Similarly, as for ${\left\|q_{u}[n]-q_{j}[n]\right\|}^{2}$, select $q_{u}^{m}[n]$ and $q_{j}^{m}[n]$ as the Taylor expansion points, the inequality relationship of Equation (40) can be obtained.

(40) $$||q_{u}[n]-q_{j}[n]|{|}^{2}\geq 2{\left(q_{u}^{m}[n]-q_{j}^{m}[n]\right)}^{T}\times (q_{u}[n]-q_{j}[n])-||q_{u}^{m}[n]-q_{j}^{m}[n]|{|}^{2},\;\forall n,u,j\neq u$$

Constraint (32b) is non-convex because the left hand side of ≥ is convex, which means we can still use the first-order Taylor expansion of $I_{u}{\left[n\right]}^{2}$ and $\Delta q_{u}^{2}$ to obtain their lower bounds. Therefore, at given points $I_{u}^{m}[n]$ and $\Delta q_{u}^{m}$ in the *m*-th iteration, we can get information as shown in Equation (41).

(41) $$I_{u}{\left[n\right]}^{2}+\frac{\Delta q_{u}^{2}}{v_{0}^{2}}\geq I_{u}^{m}{\left[n\right]}^{2}+2I_{u}^{m}[n]\left(I_{u}\left[n\right]-I_{u}^{m}\left[n\right]\right)+\frac{\Delta q_{u}^{\left(2\right)m}}{v_{0}^{2}}+\frac{2\left(\Delta q_{u}^{m}\right)\left(\Delta q_{u}-\Delta q_{u}^{m}\right)}{v_{0}^{2}}≜Q_{u}^{\text{lb},m}[n]$$

After the above processing steps, we transform all non convex constraints into convex constraints, and thus, we get the approximated solution (P8) by dealing with the problem (P9) of Equation (42).

(42a) $$\left(\mathrm{P9}\right)\;:\max_{\left\{Q,S,I,\mu \right\}}\;\mu \;s.t.\;\;\sum_{n=1}^{N-1}\sum_{u=1}^{U}\delta b_{u,k}[n]B({\overset{ˆ}{R}}_{u,k}^{\mathrm{lb}}[n]-\log_{2}\left(\sum_{i=1,i\neq k}^{K}\frac{p_{i}[n]\beta_{0}}{H^{2}+S_{u,i}[n]}+\sigma^{2}\right))\geq \eta ,\forall \;k$$

(42b) $$S_{u,i}[n]\leq ||q_{u}^{r}[n]-g_{i}|{|}^{2}+2{\left(q_{u}^{r}[n]-g_{i}\right)}^{T}\;\;\;\;\times \;(q_{u}[n]-q_{u}^{r}[n]),\;\forall \;u,n,i\neq k$$

(42c) $$d_{s}^{2}\leq 2{\left(q_{u}^{m}[n]-q_{j}^{m}[n]\right)}^{T}\times \;(q_{u}[n]-q_{j}[n])\;-||q_{u}^{m}[n]-q_{j}^{m}[n]|{|}^{2},\;\forall n,u,j\neq u$$

(42d) $${\left\|q_{u}[n+1]-q_{u}[n]\right\|}_{2}\leq D_{\max},n=1,\cdots ,N-1$$

(42e) $$q_{u}[1]=q_{u}[N],\forall u$$

(42f) $$\sum_{u=1}^{U}E_{u,uav}(\{q_{u}[n]\},\{I_{u}[n]\})+E_{GN}\leq E_{\varepsilon }$$

(42g) $$Q_{u}^{\text{lb},m}[n]\geq \frac{\delta^{4}}{I_{u}{\left[n\right]}^{2}},n=1,\cdots ,N-1$$

We can find that the constraint (42a) is convex, and (42b), (42c), (42g) are all linear constraints. Problem (P9) can be solved by some existing convex optimization solvers such as CVX.

### 3.3 Collection time optimization

With any given $p_{k}[n]$, $b_{u,k}[n]$ and $q_{u}[n]$, the time slot length *δ* is optimized by solving the subproblem (P10) of Equation (43).

(43a) $$\left(\mathrm{P10}\right)\;:\max_{\left\{\delta ,\mu \right\}}\;\mu \;s.t.\;\;E_{U}(\delta )+E_{GN}(\delta )\leq E_{\varepsilon }$$

(43b) $$\sum_{n=1}^{N-1}\sum_{u=1}^{U}\delta b_{u,k}[n]BR_{u,k}[n]\geq \mu ,\forall k$$

(43c) $${\left\|q_{u}[n+1]-q_{u}[n]\right\|}_{2}\leq \delta V_{max},n=1,\cdots ,N-1$$

In this situation, the third item of UAV flight energy consumption $E_{u}$ is non-convex with respect to *δ*. By using the slack variable $I=\{I_{u}[n]\geq 0,\forall u\}$ defined before, we have the equivalence relation shown in Equation (44).

(44) $$I_{u}[n]={\left(\sqrt{\delta^{4}+\frac{\Delta q_{u}^{4}}{4v_{0}^{4}}}-\frac{\Delta q_{u}^{2}}{2v_{0}^{2}}\right)}^{1/2},I_{u}[n]\geq 0\Leftrightarrow I_{u}{\left[n\right]}^{4}+\frac{\Delta q_{u}^{2}}{v_{0}^{2}}I_{u}{\left[n\right]}^{2}=\delta^{4}$$

Now problem (P10) can be transferred into (P11) of Equation (45).

(45a) $$\left(\mathrm{P11}\right)\;:\max_{\left\{\delta ,I,\mu \right\}}\;\mu \;s.t.\;\;\sum_{u=1}^{U}E_{u}(\delta ,\{I_{u}[n]\})+E_{GN}(\delta )\leq E_{\varepsilon }$$

(45b) $$I_{u}{\left[n\right]}^{4}+\frac{\Delta q_{u}^{2}}{v_{0}^{2}}I_{u}{\left[n\right]}^{2}\geq \delta^{4}$$

(45c) $$\sum_{n=1}^{N-1}\sum_{u=1}^{U}\delta b_{u,k}[n]BR_{u,k}[n]\geq \mu ,\forall k$$

(45d) $${\left\|q_{u}[n+1]-q_{u}[n]\right\|}_{2}\leq \delta V_{max},n=1,\cdots ,N-1$$

Select $I_{u}^{m}[n]$ as the first-order Taylor expansion point in the *m*-th iteration, and use the same method in constraint (32b), the constraint of $I_{u}[n]$ can be approximated by Equation (46).

(46) $$I_{u}{\left[n\right]}^{4}+\frac{\Delta q_{u}^{2}}{v_{0}^{2}}I_{u}{\left[n\right]}^{2}\geq \frac{\Delta q^{2}}{v_{0}^{2}}I_{u}^{m}{\left[n\right]}^{2}+I_{u}^{m}{\left[n\right]}^{4}+(4I_{u}^{m}{\left[n\right]}^{3}+2\frac{\Delta q^{2}}{v_{0}^{2}}I_{u}^{m}[n])(I_{u}[n]-I_{u}^{m}[n])$$

With Equation (46), the optimization problem for obtaining the optimal time slot is described as (P12) of Equation (47).

(47a) $$\left(\mathrm{P12}\right)\;:\max_{\left\{\delta ,I,\mu \right\}}\;\mu \;s.t.\;\;\sum_{u=1}^{U}E_{u}(\delta ,\{I_{u}[n]\})+E_{GN}(\delta )\leq E_{\varepsilon }$$

(47b) $$(4I_{u}^{m}{\left[n\right]}^{3}+2\frac{\Delta q^{2}}{v_{0}^{2}}I_{u}^{m}[n])(I_{u}[n]-I_{u}^{m}[n])\;\;+I_{u}^{m}{\left[n\right]}^{4}+\frac{\Delta q^{2}}{v_{0}^{2}}I_{u}^{m}{\left[n\right]}^{2}\geq \delta^{4}$$

(467c) $$\sum_{n=1}^{N-1}\sum_{u=1}^{U}\delta b_{u,k}[n]BR_{u,k}[n]\geq \mu ,\forall k$$

(47d) $${\left\|q_{u}[n+1]-q_{u}[n]\right\|}_{2}\leq \delta V_{max},n=1,\cdots ,N-1$$

Now all constraints of problem (P12) can be solved by toolbox and obtain the sub-optimal *δ*. Finally the total optimal data collection time $T_{f}$ is obtained by $T_{f}=(N-1)\delta$.

### 3.4 Overall algorithm

Based on the processing process in the previous subsections, combined with BCD and SCA methods, we proposed a new iterative algorithm to solve problem (P1). Specifically, the original optimization problem (P1) is divided into four blocks, i.e., {B,P,Q,δ}. Then, in each iteration the data transmit scheduling **B**, transmit power **P**, UAVs trajectories **Q** and time slot *δ*, which need to be optimized alternately by solving problem (P3), (P4), (P9) and (P12) correspondingly. Moreover, the solution obtained from each iteration can be used as Taylor expansion points as input for the next iteration. The detail of our algorithm is shown at Algorithm 1.

**Algorithm 1:**
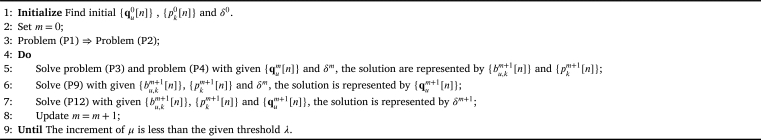
Algorithm for Problem (P1).

Firstly, for the convergence of the above algorithm, since SCA method can guarantee monotonic convergence. Secondly, as for the computational complexity of Algorithm 1, all four subproblems that need to be solved in Algorithm 1 are convex problems. Besides, the upper bound of the time complexity for solving a convex problem with CVX is $O(\sqrt{I})$, where *I* represents the quantity of inequality constraints. According to problems (27), (28), (42) and (47), the quantity of inequality constraints grows with the order of N⋅M⋅K. Hence, the complexity of Algorithm 1 can be represented as T(N,M,K)=O(N⋅M⋅K), which is in polynomial order.

## 4 Simulation results

We demonstrate the effectiveness of our algorithm by a systematic numerical simulation experiment. Multiple simulation experiments are designed for performance comparison.

• Data transmit scheduling: Set different numbers of UAVs and GNs, and compare the results of data transmit scheduling.
• Comparison of different trajectory optimization schemes: 2 UAVs with 6 GNs and 3 UAVs with 60 GNs are set up respectively, and the trajectories of multiple UAVs under different optimization schemes are compared.
• Performance comparison: Under different energy consumption constraints, the time of data collection under different trajectory optimization algorithms is compared, and the minimum data amount of nodes achieved under different trajectory optimization algorithms is compared.

### 4.1 Parameters setting

We set all UAVs are flying in a rectangular area of 2000 *m* × 2000 *m*, and all GNs are randomly distributed within this region. All UAVs' flying altitude *H* is fixed at 100 *m*. Simulation precision λ=0.005. The initial value δ=0.5s, and N=200. According to the reference [35], the rest of the relevant parameters setting are listed in Table 1.

**Table 1:** Parameters setting.

Notation	Meaning	Value
*P*_*max*_	GN transmit power	1000 mW
*V*_*max*_	UAV maximum speed	30 m/s
*B*	Channel bandwidth	1 MHz
*σ*^2^	the additive Gaussian white noise power	-110 dBm
*β*_0_	Power gain at *d*_0_=1 m	-60 dB
*U*_*tip*_	the tip speed of the rotor blade	120 m/s
*ρ*	The air density	1.225 kg/m^3^
*v*_0_	the mean rotor induced velocity in hover	4.03 m/s
*P*_0_	the blade profile power	79.86 W
*P*_*i*_	the induced power	88.63 W
*s*	The rotor solidity	0.05
*A*	The rotor disk area	0.503 *m*^2^

### 4.2 Data transmit scheduling

**Figure 3:**
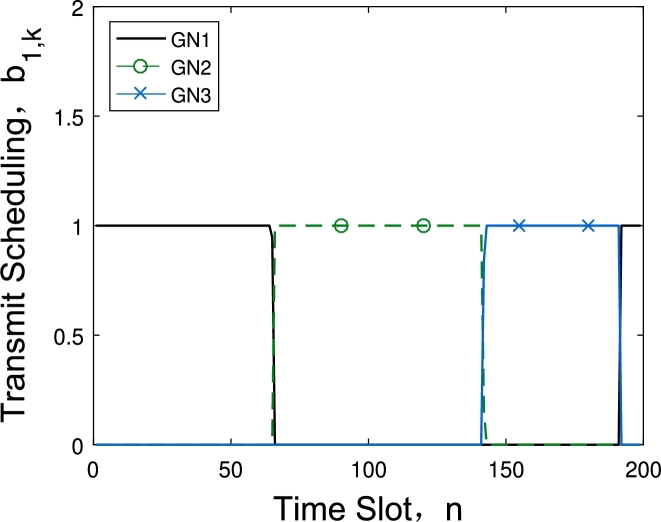
Scheduling under *E*_*ε*_ = 40 kJ.

**Figure 4:**
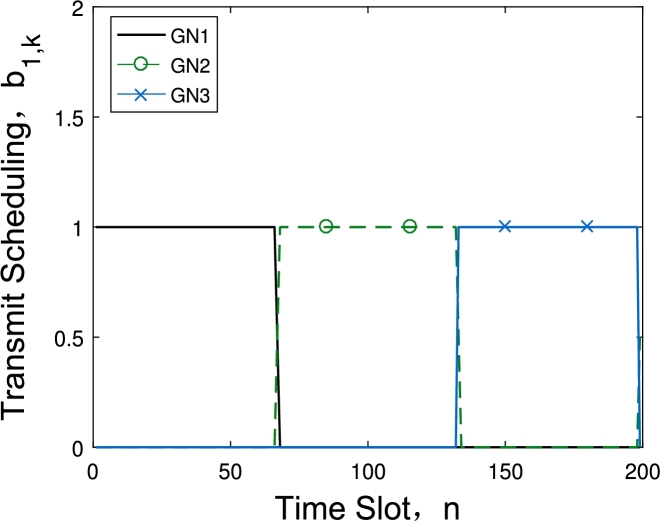
Scheduling under *E*_*ε*_ = 160 kJ.

Fig. 3 and Fig. 4 show the data collection scheduling under different energy consumption constraints with 6 GNs and 2 UAVs. Fig. 3 describes the communication scheduling of the three GNs assigned to the first UAV at energy budget is 40 kJ. We can observe that although the UAV collect data one GN after another, the duration time for different GN is not the same. Because as the distance between GN and UAV's trajectory increases, the time of UAV acquiring the GN's data will increase too, and the 3-th GN has the shortest data collection time. While in Fig. 4, the energy budget has increased to 160 kJ, and this value is already large enough and the UAV can fly directly above each node to acquire data. The time for the UAV to collect the data of each node is almost the same.

**Figure 5:**
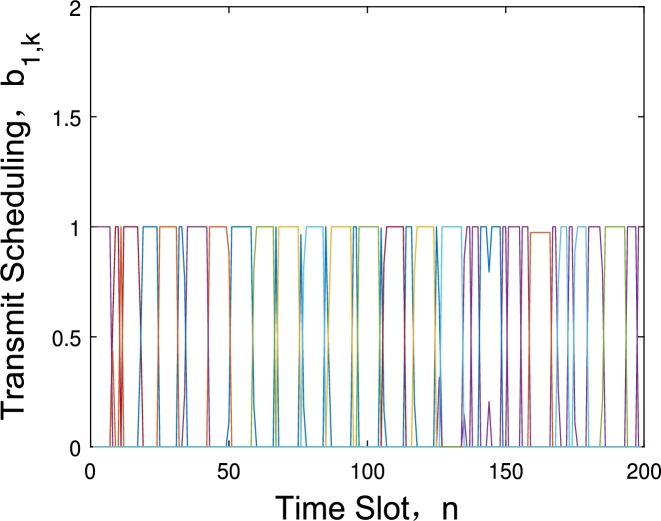
Data collection scheduling for 20 ground nodes.

Fig. 5 shows the data transmit scheduling when the number of GN increases to 20. Similar to Fig. 3, it can be seen that different nodes have different data collection times, and the nodes with longer transmission time are farther from the UAV.

**Figure 6:**
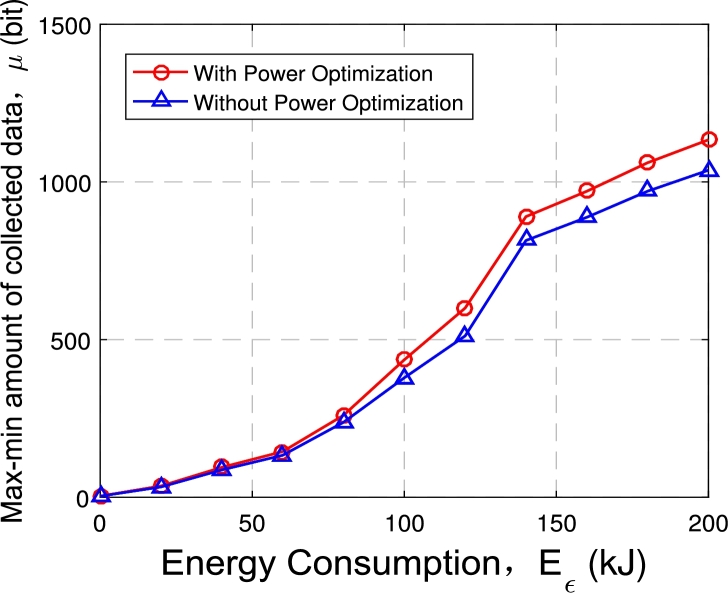
Max-min *μ* with and without power optimization.

Fig. 6 illustrates the optimization object value *μ* for both cases with and without the power optimization scheme. Under the two schemes, the optimization objective *μ* increases as the energy consumption constraint becomes larger, and the increase speed is faster when the energy consumption constraint is small. Then, the minimum amount of data collected on ground nodes with transmit power optimization is increased by 5% to 15% compared to when there is no transmit power optimization.

### 4.3 Multi-UAV trajectories comparison

In this subsection, the multi-UAV trajectory of 2 UAVs and 6 nodes is compared with that of 3 UAVs and 60 nodes.

**Figure 7:**
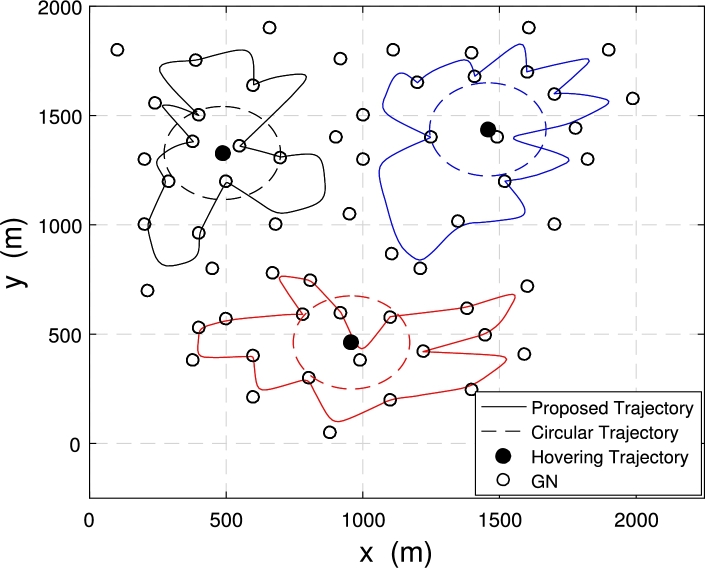
Trajectories under *E*_*ε*_ = 120 kJ.

**Figure 8:**
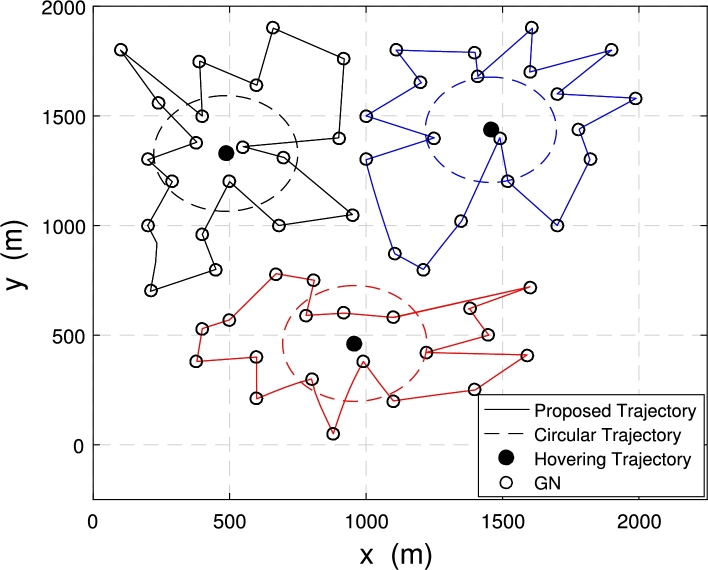
Trajectories under *E*_*ε*_ = 300 kJ.

Fig. 7 and Fig. 8 show the comparison of the proposed trajectories with the baseline trajectories when 3 UAVs with 60 nodes are deployed in the system.

Fig. 7 shows the trajectory comparison of multiple UAVs when the energy consumption constraint $E_{\varepsilon }=120$ kJ. The energy consumption constraint is not enough to allow the three UAVs to fly directly above each node to collect data. The proposed optimized trajectories are still getting close to the node as possible to obtain higher data transmit speed and achieve more data collection from GNs. At this time, the minimum data amount of ground nodes collected by the multi-UAV is μ=63.17 bit with proposed trajectories.

Fig. 8 illustrates the UAV trajectories achieved with different trajectory optimization schemes when the system energy consumption constraint is $E_{\varepsilon }=300$ kJ. The energy consumption constraint is large enough. The proposed optimized trajectories can make each UAV fly directly above each node to collect data. At this time, the distance between the UAVs and the nodes is the shortest, and the data collection rate is the fastest, which can achieve more amount. At this time, the maximum data volume of the node is μ=220.51 bit, which achieved more amount of collected data compared to the situation in Fig. 7. Also, data collection time under this case is $T_{f}=605.24$.

**Figure 9:**
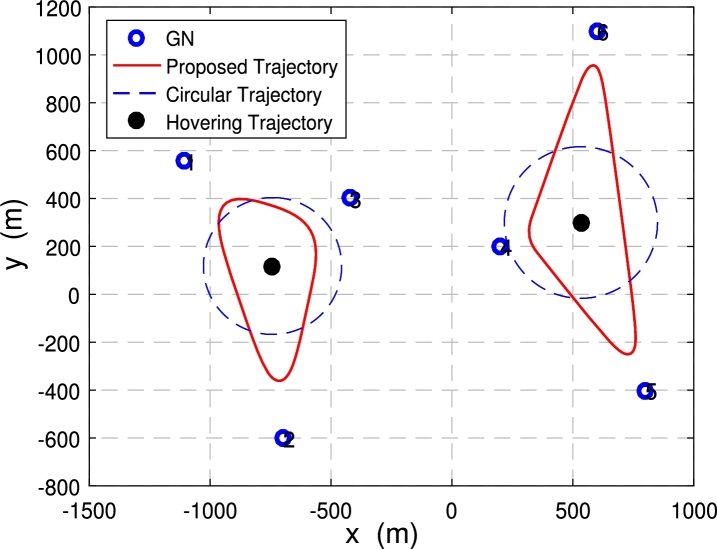
Trajectories comparison with 2 UAVs and 20 GNs.

Fig. 9 shows three different trajectory optimization schemes when deploying 2 UAVs with 6 nodes with $E_{\varepsilon }=40$ kJ. In Fig. 9, the given energy consumption constraint is relatively small, 2 UAVs do not have enough energy to directly collect data, but the proposed optimized trajectory is still trying to make UAVs as close to each ground node as possible to achieve faster data transmit rate.

**Figure 10:**
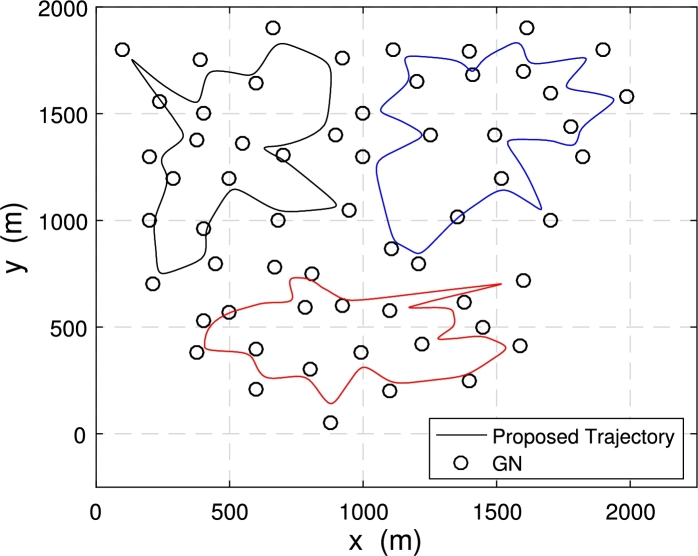
Proposed trajectories under *E*_*ε*_ = 300 kJ with *μ*_*max*_ = 300 bit.

Now we deploy 3 UAVs and 60 nodes in the system, the energy consumption is limited to $E_{\varepsilon }=300$ kJ, and the maximum value of *μ* is set to $\mu_{max}=300$ bit. Fig. 10 illustrates the multi-UAV optimization trajectory achieved by the proposed scheme.

In this case, UAVs do not need to achieve the maximum amount of data collection, so compared to Fig. 8, the trajectory of each drone is shorter and consumes less flight energy and the corresponding time to complete data collection is $T_{f}=420.37$, which is also shorter than the time in Fig. 8.

### 4.4 Performance comparison

#### 4.4.1 Data collection time

**Figure 11:**
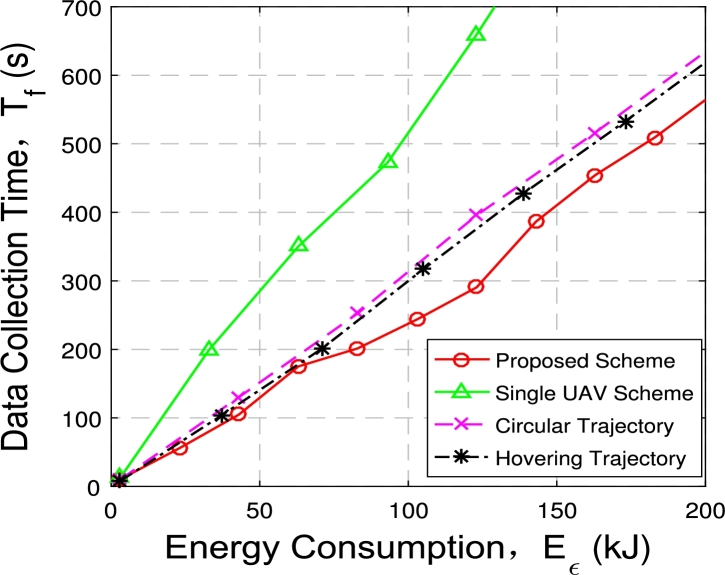
Data acquisition time with 2 UAVs and 6 GNs.

**Figure 12:**
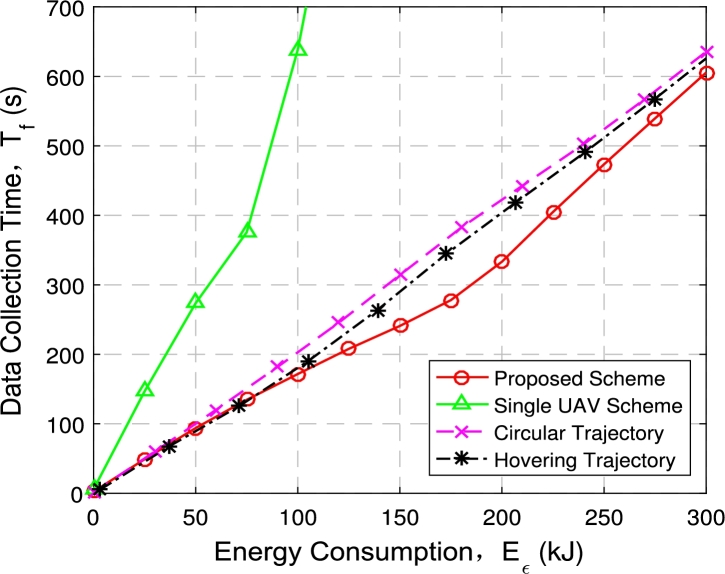
Data acquisition time with 3 UAVs and 60 GNs.

Fig. 11 and Fig. 12 illustrate the relationship between data collection time and power consumption constraints in a multi-UAV data collection system.

Fig. 11 shows the comparison of data collection time under four optimization schemes when 2 UAVs and 6 ground nodes are deployed: (1) the proposed multi-UAV scheme; (2) the single-UAV scheme; (3) multi-UAV hovering scheme; (4) multi-UAV circular scheme.

We can observer that the time of the four schemes increases with energy consumption constraint. Data collection time of the three multi-UAV trajectory schemes are relatively similar when the energy consumption constraints are small. At this condition, trajectories under three multi-UAV schemes are relatively similar, therefore the data collection time is relatively similar. When the given energy consumption constraint is large enough, the proposed multi-UAV trajectory optimization scheme can make the UAVs closer to each node, and the speed of UAVs is faster, hence the data collection time is shortest among all schemes. In addition, two UAV systems can save about 50% of the time compared to single UAV system under the same energy consumption constraints.

Fig. 12 illustrates the comparison of data collection time under the four schemes when deploying 3 UAVs and 60 nodes. When the number of UAVs and nodes increases, the growth trend of data collection time is consistent with that in Fig. 11, and it increases with energy consumption constraint. The data collection time under the proposed multi-UAV optimization scheme is shorter than other schemes. In addition, three UAVs can save about 66.67% of the time compared to single UAV. This further illustrates the superiority of deploying multi-UAV in reducing data collection time.

#### 4.4.2 Minimum data collected

**Figure 13:**
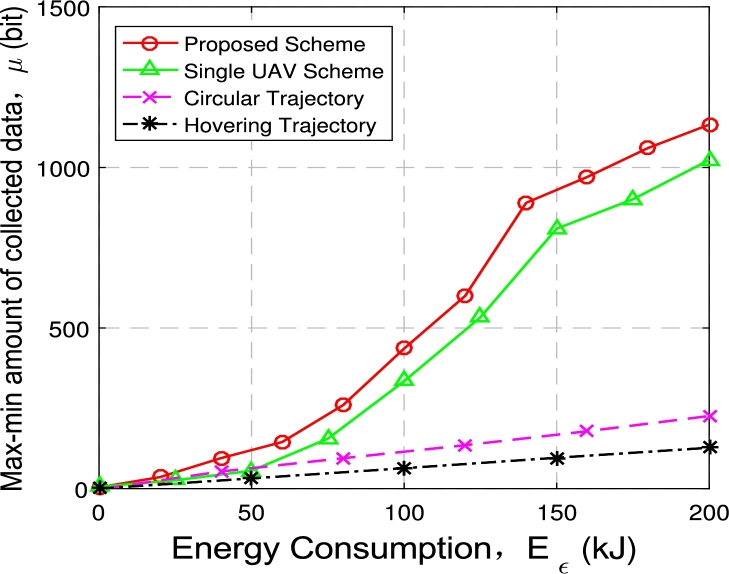
Max-min *μ* with 2 UAVs and 6 GNs.

**Figure 14:**
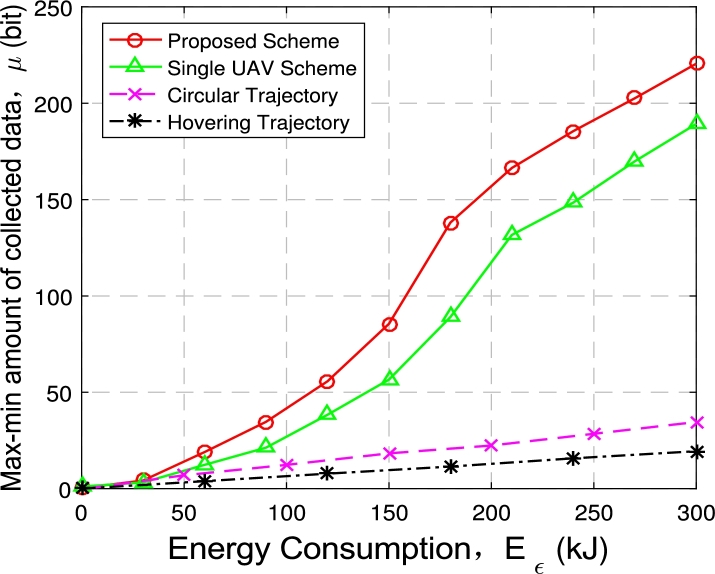
Max-min *μ* with 3 UAVs and 60 GNs.

Fig. 13 and Fig. 14 shows the relationship between the minimum amount of data collected from ground nodes and power consumption constraints, and compares the performance under four schemes: (1) the proposed multi-UAV scheme; (2) the single-UAV scheme; (3) multi-UAV hovering scheme; (4) multi-UAV circular scheme.

Fig. 13 illustrates the minimum amount of data collection when deploying 2 UAVs and 6 nodes. With the increase of the power consumption constraint $E_{\varepsilon }$, the minimum data amount of data collected under four optimization schemes also increases.

For the proposed optimization scheme, when $E_{\varepsilon }\leq 140$ kJ, as the data collection time increases, the distance between nodes and UAVs trajectories shortens, and the value of the optimization objective *μ* increases faster. When $E_{\varepsilon }\geq 140$ kJ, the energy consumption constraint is large enough to allow UAVs to collect data directly above each node. Therefore, the increase in energy consumption leads to an increase in data collection time, while the minimum distance does not change. Hence the optimization objective *μ* showed a linear growth trend.

The optimization objective *μ* achieved by the proposed optimization scheme is significantly larger than the other three optimization schemes. In addition, comparing two UAVs with single UAV, the increase in the minimum data amount *μ* collected from ground nodes is 15% to 25%.

Fig. 14 illustrates the minimum amount of data collection when deploying 3 UAVs with 60 nodes. With more UAVs and nodes, the growth trend of the node minimum amount of collected data *μ* achieved by the four optimization methods is roughly the same as in Fig. 13. When the given energy consumption constraint is small, the proposed optimization scheme can achieve a faster growth rate of the minimum data collection amount *μ*. When the energy consumption constraint is large enough, the optimization objective *μ* achieved by the proposed optimization scheme also increases approximately linearly.

Comparing the circular scheme and the hovering scheme, the proposed optimization scheme achieves more minimum data collection *μ*. In addition, when the number of nodes is 60, the optimization target *μ* is significantly improved when three UAVs are compared with single UAV.

Combined with Fig. 13 and Fig. 14, the proposed multi-UAV optimization scheme has obvious advantages compared with the baseline schemes in maximizing the minimum data amount *μ* of ground nodes, and it also has a considerable performance improvement compared with the single UAV situation, and it also significantly reduces data collection time. Under the same energy consumption constraints, the proposed multi-UAV optimization scheme can collect more data from ground nodes in less time than the three benchmark optimization schemes.

## 5 Conclusion

In this paper, we consider a data collection system assisted by multiple UAVs under the constraint of total energy consumption of the system, which includes the flight energy consumption of all UAVs and the energy consumption for data transmission for all GNs. Then, UAVs trajectories, GN's transmit power, data collection scheduling and data collection time joint design problem was proposed to maximize the minimum amount data collected by all UAVs from each GN. To solve the above non convex problem, we designed an iterative algorithm based on BCD and SCA method. Finally, the numerical simulation results show that our proposed method is more effective compared to other baseline schemes and can collect more data from GNs under the same energy budget. In our future work, expanding our model to more complex and realistic communication channel scenarios and considering 3D trajectory optimization are very attractive.

## CRediT authorship contribution statement

**Qing Cai:** Conceptualization, Data curation, Formal analysis, Funding acquisition, Investigation, Methodology, Project administration, Resources, Software, Supervision, Validation, Visualization. **Zheng Tang:** Formal analysis, Investigation, Methodology, Validation, Visualization, Writing – original draft, Writing – review & editing. **Chuan Liu:** Conceptualization, Data curation, Formal analysis, Funding acquisition, Investigation, Methodology, Project administration, Resources, Software, Supervision, Validation, Visualization, Writing – original draft.

## Declaration of Competing Interest

The authors declare that they have no known competing financial interests or personal relationships that could have appeared to influence the work reported in this paper.

## Data Availability

The data that has been used is confidential.
